# Trends over Time of Lung Function and Radiological Abnormalities in COVID-19 Pneumonia: A Prospective, Observational, Cohort Study

**DOI:** 10.3390/jcm10051021

**Published:** 2021-03-02

**Authors:** Pierachille Santus, Nicola Flor, Marina Saad, Stefano Pini, Elisa Franceschi, Andrea Airoldi, Paolo Gaboardi, Sonia Ippolito, Maurizio Rizzi, Dejan Radovanovic

**Affiliations:** 1 Department of Biomedical and Clinical Sciences (DIBIC), Università degli Studi di Milano, 20157 Milano, Italy; pierachille.santus@unimi.it (P.S.); marina.saad@unimi.it (M.S.); stefano.pini@unimi.it (S.P.); elisa.franceschi@unimi.it (E.F.); paolo.gaboardi@unimi.it (P.G.); 2 Division of Respiratory Diseases, Ospedale Luigi Sacco, Polo Universitario, ASST Fatebenefratelli-Sacco, 20157 Milano, Italy; andrea.airoldi@asst-fbf-sacco.it (A.A.); maurizio.rizzi@asst-fbf-sacco.it (M.R.); 3 Division of Radiology, Ospedale Luigi Sacco, Polo Universitario, ASST Fatebenefratelli-Sacco, Via G.B. Grassi 74, 20157 Milano, Italy; nicola.flor@asst-fbf-sacco.it (N.F.); sonia.ippolito@asst-fbf-sacco.it (S.I.)

**Keywords:** COVID-19, lung function, lung diffusion, D-dimer, DLCO, spirometry, chest CT

## Abstract

Radiological and functional sequelae of Coronavirus Disease 2019 (COVID-19) pneumonia are still poorly understood. This was a prospective, observational, physiological, cohort study on consecutive adult patients with COVID-19 pneumonia admitted in April–May 2020 in the high dependency respiratory unit of L. Sacco University Hospital in Milan (Italy). During hospitalization, patients underwent chest computed tomography (CT), blood gas analysis, spirometry, and lung diffusion capacity for carbon monoxide (DLco), which were repeated 6 weeks post-discharge. Chest CTs were individually read by two expert radiologists, that calculated the total severity score (TSS). Twenty patients completed the study (mean age 58.2 years, 70% males). During the acute phase, mean DLco, alveolar volume (VA), and vital capacity (VC) were 56.0 (16.3), 64.8 (14.0), and 71.7 (16.9) % predicted, respectively, and were inversely associated with PaO_2_/FiO_2_ ratio. Fifty percent of patients had a restrictive ventilatory pattern; mean TSS was 7.9 (4.0). At follow up, gas exchange parameters were normalized; consolidations persisted in 10% of cases, while DLco was <80% predicted in 65% of patients and was independently predicted by Log_10_D-dimer at admission (β −18.675; 95%CI, −28.373–−9.076; *p* = 0.001). In conclusion, functional abnormalities in COVID-19 pneumonia survivors can persist during follow up and are associated with the severity of the disease.

## 1. Introduction

Since the initial outbreak in Wuhan (China), the Coronavirus Disease 2019 (COVID-19) pandemic has caused more than 1,200,000 deaths worldwide [1], leading to an unprecedented burden on healthcare systems with hundreds of thousands patients needing hospitalization [2]. The lung damage caused by COVID-19 is characterized by a variable spectrum of interstitial, organizing, and acute fibrinous organizing pneumonia [3], reflecting ground glass opacities, crazy paving patterns, and pulmonary consolidations at the chest computed tomography (CT) [4]. In addition, hypoxic respiratory failure can be worsened by endothelial dysfunction and a pro-thrombotic diathesis that sustain a worsening of ventilation/perfusion mismatch and are responsible for increased risk for adverse outcomes in patients with severe disease [3,5,6]. The radiological evolution of COVID-19 pneumonia during the acute phase of the disease has been extensively studied [7,8], but the pathophysiological and clinical impact of potential pulmonary sequelae is still under investigation. Some line of evidence suggested that thirty days from discharge the CT abnormalities resolve almost completely in mild to moderate COVID-19 forms [9]. Other Authors, however, reported that in patients with moderate, severe and critical disease, both ground glass opacities and septal thickening tend to persist up to three months post-discharge [8,10,11,12,13,14]. Accordingly, the few available studies conducted on recovering COVID-19 patients have shown that the main lung function complications are represented by reduced lung diffusion capacity for carbon monoxide (DLCO) and, more rarely, by impaired static and dynamic volumes [10,11,12,13,15,16]. These observations are in line with previous studies conducted in Severe Acute Respiratory Syndrome Coronavirus (SARS-CoV) [17,18] and Middle East Respiratory Syndrome Coronavirus (MERS-CoV) survivors [19]. However, differently from what was observed in SARS survivors [18], in convalescent COVID-19 patients lung function parameters were scarcely associated with the extension and quality of CT abnormalities [10,11]. Indeed, so far, the lung function pattern observed during follow up did not find a satisfactory explanation [10,15,16], the greatest limitation being the lack of previous lung functional assessments to be compared with [10,15,16].

By prospectively assessing lung function, gas exchange, and chest CT findings both during the acute phase and during follow up, we aimed at investigating the pulmonary pathophysiology and the short-term complications of COVID-19 pneumonia and at exploring the possible relationship between the evolution of functional abnormalities, gas exchange and radiological sequelae.

## 2. Materials and Methods

This was an observational, prospective, monocentric, physiological, cohort study, conducted from April to July 2020 in the high dependency respiratory care unit (HDRU) of Luigi Sacco University Hospital in Milan (Italy).

The study protocol (ClinicalTrials. gov: NCT04307459) followed the amended Declaration of Helsinki (2013), was approved by the local ethical committee (Comitato Etico Milano Area I; 17263/2020), and all recruited patients gave written informed consent.

Adult hospitalized patients with a virologically confirmed diagnosis of SARS-CoV-2 infection [20,21] were considered eligible for the study and consecutively enrolled from April to May 2020 (for details on COVID-19 diagnosis, please see Supplementary Materials). Inclusion criteria were: 1) age ≥ 18 years old; and 2) confirmed diagnosis of SARS-CoV-2 pneumonia. Patients were excluded from the study if unable to provide the informed consent or to perform repeatable lung function tests.

**Figure 1:**
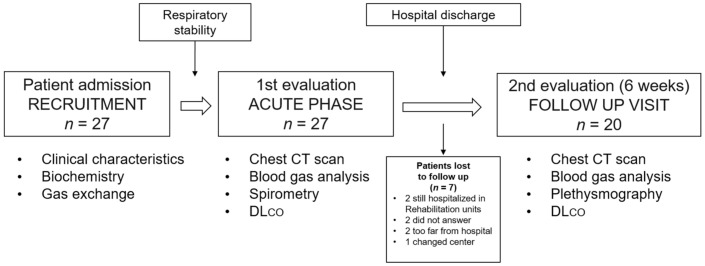
Flow chart of the study protocol. Seven patients were lost to follow up. DLco: lung diffusion capacity for carbon monoxide.

Anthropometrical, clinical characteristics, gas exchange parameters, and biochemistry were collected at admission. As soon as clinical stability was reached, while still hospitalized in the HDRU, patients underwent chest CT, blood gas analysis, spirometry, and DLco in the same day (Figure 1). The same routine was executed at the follow up visit, scheduled at 6 weeks (± 5 days) post-discharge (Figure 1). In that instance, body-plethysmography was performed instead of spirometry. At follow up, all patients had a negative SARS-CoV-2 nasopharyngeal swab, and lung function maneuvers were performed in the respiratory outpatient clinic as suggested by national and international safety recommendations [22,23].

### 2.1. Outcomes

The primary objective was to investigate changes in lung function parameters (static, dynamic volumes, and DLco), gas exchange, and chest CT findings assessed during hospitalization and after 6 weeks from hospital discharge.

Secondary objectives were: (1) to assess the relationship between gas exchange, lung function abnormalities and radiological patterns during hospitalization and during the recovery phase; and (2) to explore the determinants of DLco impairment during follow up.

### 2.2. Procedures

Gas exchange parameters were obtained from an arterial blood sample at rest. Details on gas exchange parameters and criteria used to grade respiratory failure are reported in the Supplementary Materials. During hospitalization, patients underwent lung function testing with spirometry and DL_CO_ as soon as clinical stability was achieved. Clinical stability was considered when patients were able to tolerate a disconnection from oxygen therapy for the duration of the test and met the following criteria: SpO_2_ ≥ 94% on ≤ 50% FiO_2_; no need for continuous invasive or non-invasive respiratory support; absence of respiratory distress at rest; arterial systolic pressure ≥ 100 mmHg and diastolic pressure ≥70 mmHg. An obstructive pattern was considered a forced expiratory volume in one second (FEV_1_)/vital capacity (VC) ratio < Lower Limits of Normal (LLN) [24], and a restrictive pattern was a total lung capacity (TLC) < 80% predicted [24]. As plethysmography was available only during follow up, a pattern suggestive for restriction was defined as a FEV_1_/VC ≥ 100% predicted associated with a FEV_1_ and VC < 80% predicted. Lung function assessments were performed according to current American Thoracic Society/European Respiratory Society (ATS/ERS) recommendations [24,25] and are detailed in Supplementary Materials.

Multidetector chest CT was performed using either a 64-MDCT Lightspeed scanner (GE Medical Systems, Milwaukee, WI, USA) or a 64-MDCT Brilliance scanner (Philips Medical System, Amsterdam, Netherlands). Two experienced radiologists (N.F. and S.I.) retrospectively and independently reviewed the images on a PACS workstation (IMPAX, Agfa Healthcare, Mortsel, Belgium). Chest CT images were assessed for the presence of peripheral and multifocal ground glass opacities, consolidations, fibrosis, and crazy-paving patterns. The severity of disease was evaluated using the Total Severity Score (TSS), as previously reported [26,27]. CT scanning protocols and scoring method is reported in the Supplementary Materials.

Respiratory failure and pharmacological therapy were managed according to standard operating procedures and available recommendations [22,23,28,29,30,31,32] (Supplementary Materials).

### 2.3. Statistics

The sample size was determined by the dynamics of the COVID-19 pandemic, and recruitment was stopped when the COVID-19 HDRU was closed in May 2020. At the time of the study, no evidence on changes in lung function parameters in patients with COVID-19 pneumonia was available, so it was not possible to power the study. Qualitative variables were presented as absolute and relative (percentage) frequencies. The Shapiro Wilk test was used to assess normality of data distribution. Parametric and non-parametric quantitative variables were described with means (standard deviation, SD) and medians (inter-quartile ranges, IQRs), respectively. McNemar’s test was used to compare qualitative variables, while Student’s t-test, Wilcoxon or Mann–Whitney U test were used to compare quantitative variables with normal or non-normal distribution, respectively. Relationships between variables were assessed by means of linear regression analysis. Predicted normal values of spirometric, plethysmographic, and DLco parameters were derived from Quanjer [33]. Clinical, functional, and radiological characteristics of patients with preserved (≥ 80% predicted) and impaired (<80% predicted) DLco were compared to assess predictors for DLco during follow up. Significant variables at the univariate analysis were used for the multiple regression analysis. The D-dimer value was log10 transformed to fit a normal distribution. A two-tailed *p*-value < 0.05 was considered statistically significant. All statistical computations were performed with “IBM SPSS Statistics for Windows”, version 23 (IBM Corp, Armonk, NY, USA).

## 3. Results

A total of 27 patients were enrolled in the study, and 7 were lost at the follow up visit. Details about the reasons for drop-out are reported in the Supplementary Materials. Twenty patients (mean age 58.2, 70% males) were included in the final analysis. Anthropometrical and clinical characteristics are reported in Table 1. The most frequent comorbidity was arterial hypertension (55%), 70% of patients were lymphopenic, and the median (IQR) D-dimer at admission was 768 (374–3966) mg/L fibrinogen equivalent units (FEU). Nine patients (45%) had a D-dimer > 1000 mg/L FEU. The mean (SD) PaO_2_/FiO_2_ ratio at admission was 264 (128) mmHg. A-a gradient was increased in all patients, and 95% had an A-a gradient > 100 mmHg. Continuous Positive Airway Pressure (CPAP) was employed in 13 patients (65%); four of them eventually required invasive mechanical ventilation and were subsequently re-admitted in the HDRU once weaned from invasive mechanical ventilation (IMV). The first lung function assessment was performed on average 28.2 (13.5) days from symptoms onset and 9.1 (6.3) from admission (Table 1), while the follow up assessment was performed 43.8 (5.7) days from hospital discharge. Four patients were discharged on oxygen therapy and 12 (60%) continued low molecular weight heparin (LMWH) after discharge.

### 3.1. Gas exchange and Lung Function

At the time of first evaluation, the mean PaO_2_/FiO_2_ and median A-a O_2_ gradient were 316 (87) and 34 (19–101) mmHg, respectively. At the follow up visit, none of the patients had respiratory failure, and the A-a O_2_ gradient was normalized in all patients (Table 2).

**Figure 2:**
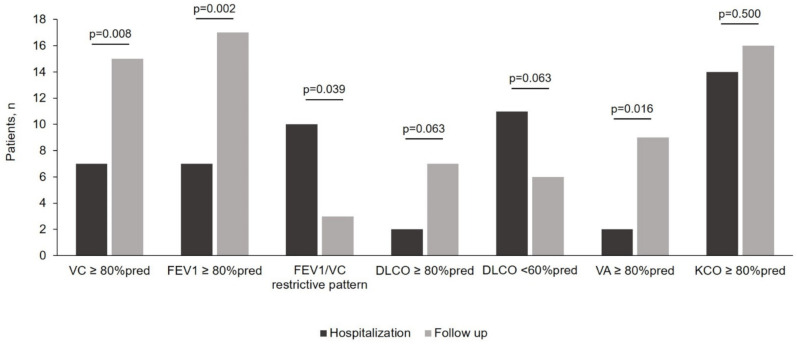
Change in the proportion of patients with lung function alterations during hospitalization (dark grey columns) and at the follow up visit (light gray columns-6 weeks post-discharge). Restrictive pattern was considered the combination of a FEV_1_/VC ≥ 100% predicted and a concomitant FEV_1_ and VC < 80% predicted. DLco: lung diffusion capacity for carbon monoxide; FEV_1_= forced expiratory volume in one second; KCO = transfer factor for carbon monoxide; VA = alveolar volume; VC = vital capacity.

During hospitalization, VC and FEV_1_ were equally reduced (Table 2), which justified a median FEV_1_/VC ratio of 104.5 (98.2–114.0) % predicted. Accordingly, 9 (45%) patients had a spirometry suggestive for a restrictive pattern (Figure 2). DLco was reduced in 18 (90%) patients, and moderately/severely reduced in 10 patients (50%), mostly secondary to a reduction in VA (90% of patients) (Figure 2). At the follow up visit, all dynamic volumes improved (Table 2) and the FEV_1_/VC ratio remained unchanged, with 7 patients with persistent restrictive pattern. DLco improved from 56 (16.3) % predicted to 67.2 (18.0) % predicted (*p* < 0.001), but the proportion of patients with normal DLco and VA increased from 2 to 7 (*p* = 0.063) and from 2 to 9 (*p* = 0.016), respectively (Table 2 and Figure 2).

### 3.2. Chest CT

**Figure 3:**
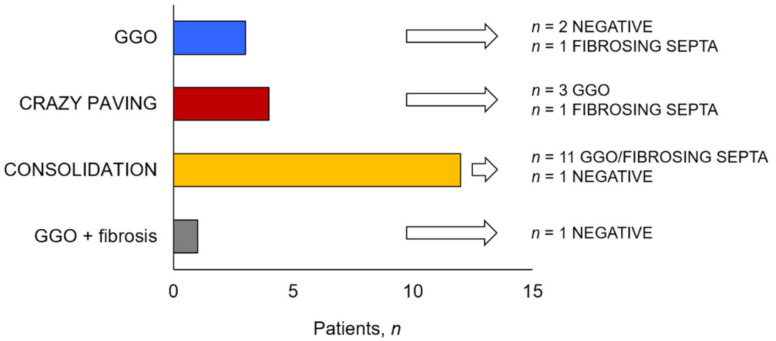
Changes in radiological patterns after 6 weeks from hospital discharge. GGO = ground glass opacities.

The chest CT performed during hospitalization showed peripheral ground glass (pGGO), consolidation and fibrosis in 70%, 65%, and 60% of patients, respectively. The mean TSS was 7.9 (4.0) (Supplementary Materials, Table S1). At 6 weeks post-discharge, consolidations were still present in only 2 patients (10%), while the proportion of CT scans showing multifocal ground glass (mGGO) increased from 30% to 80% (*p* = 0.002) (Figure 3). At follow up, TSS was numerically reduced (mean difference (95%CI): 1.6 (−3.7–0.46); *p* = 0.118).

### 3.3. Relationship between Lung Function, Radiological Patterns and Gas Exchange

**Figure 4:**
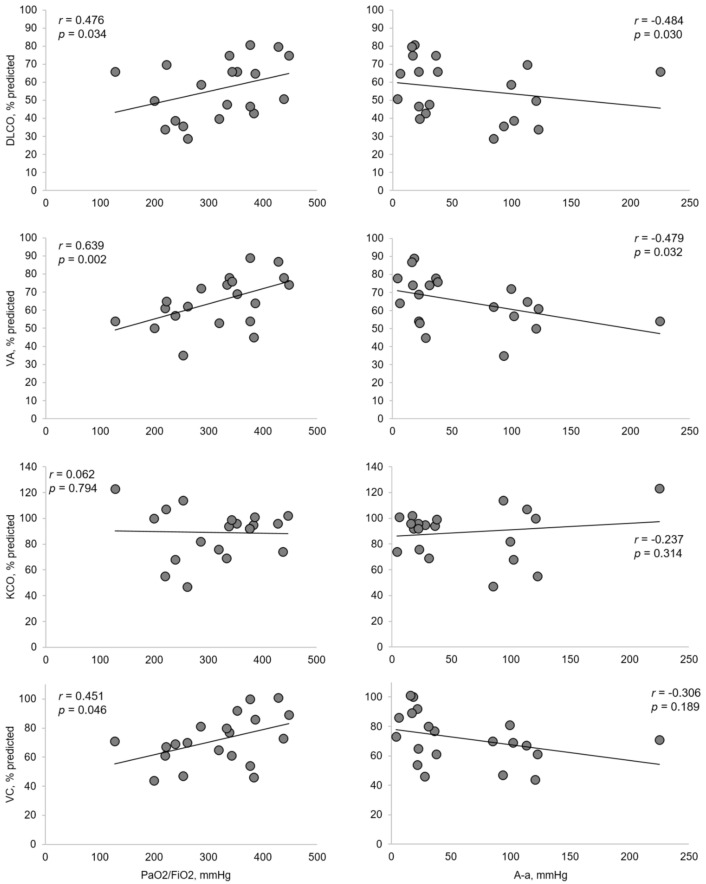
Relationship between PaO_2_/FiO_2_ ratio, A-a O_2_ gradient and lung diffusion parameters and VC. Correlations with FVC and FEV_1_ are not shown due to the strong association with VC (r = 0.859 and r = 0.829, respectively) and between FVC and FEV_1_ (r = 0.884). DLco: lung diffusion capacity for carbon monoxide; VA = alveolar volume; KCO = transfer factor for carbon monoxide; VC = vital capacity.

**Figure 5:**
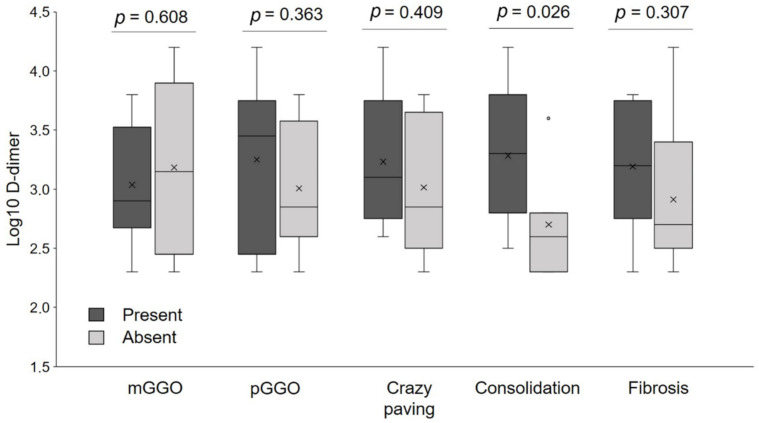
Distribution of D-dimer at admission in patients with different radiological pattern at the chest computed tomography (CT) performed at the time of first evaluation. Note that the only significant difference in D-dimer was in patients with and without parenchymal consolidations. Vertical lines represent the 75th and 25th percentile. Horizontal bars are medians. X signs indicate means. pGGO = peripheral ground glass opacities; mGGO = multifocal ground glass opacities.

The presence of crazy paving and consolidations during the hospitalization was negatively correlated with ventilation inhomogeneity (*p* = 0.021), while consolidations were negatively correlated with DLco (*p* = 0.032) and VA (*p* = 0.011) (Supplementary Materials, Table S2). At the follow up CT, the persistence of consolidations negatively correlated with dynamic volumes and lung diffusion parameters (Supplementary Materials, Table S2). Severity of respiratory failure was positively correlated with DLco, VA, and VC (Figure 4). D-dimer values at admission were higher in patients with consolidations and were not associated with other chest CT abnormalities (Figure 5).

### 3.4. Determinants of DLco during Follow up

**Figure 6:**
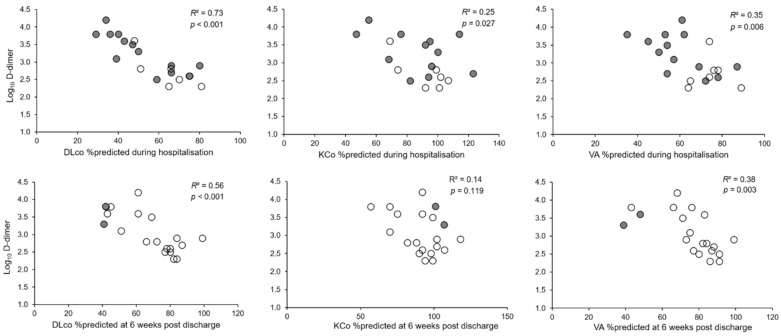
Relationship between D-dimer values at admission and DLco, KCO, and VA % predicted at first assessment during hospitalisation (upper panels) and after 6 weeks from hospital discharge (lower panels). Full circles are patients with consolidations at the chest CT perfromed the same day of the DLco, and empty circles indicate patients without consolidations. DLco = lung diffusion capacity for carbon monoxide; KCO = transfer factor for carbon monoxide; VA = alveolar volume.

**Figure 7:**
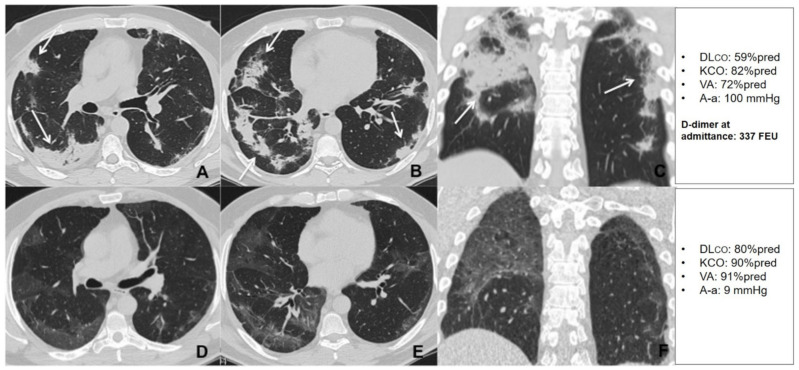
Unenhanced thin section axial (**A**,**B**) and coronal (**C**) CT images of the lungs obtained the 10th day post-admission in a 51-year-old man with no previous exposure to cigarette smoke, no cormorbidities, that was admitted to the high dependency respiratory care unit (HDRU) with severe respiratory failure (PaO_2_/FiO_2_ 182 mmHg) and treated with continuous positive airway pressure (CPAP) for 10 days. The images show peripheral consolidations (arrows) in both lungs, with predilection for posterior areas. Unenhanced thin section axial (**D**,**E**) and coronal (**F**) CT images of the same patient 6 weeks post-discharge show bilateral peripheral GGO with resolution of previous consolidations. The patient had a low D-dimer at admission and showed a significant improvement in his DLco during the recovery period.

**Figure 8:**
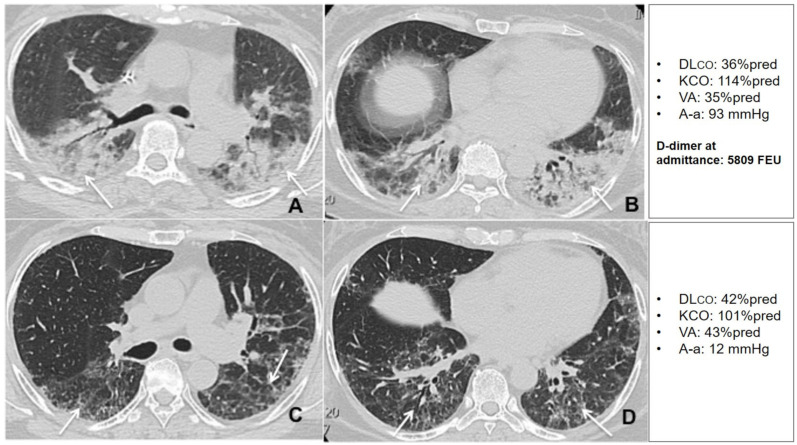
Unenhanced thin section axial (**A**,**B**) CT images of the lungs obtained the 12th day post-admission in a 57-year-old woman with arterial hypertension and no previous exposure to cigarette smoke, that was admitted to the HDRU with a PaO_2_/FiO_2_ of 210 mmHg, rapidly worsened her respiratory status and needed invasive mechanical support for 8 days. The images show peripheral posterior GGO and consolidations in both lungs, with predilection for posterior areas (arrows). Unenhanced axial (**C**,**D**) CT images of the same patient 6 weeks post-discharge show residual interlobular septal thickening with intralobular lines (arrows) with resolution of previous GGO and consolidation. The patient had a high D-dimer at admission and showed very limited changes in DLco during the recovery period.

When compared with patients with preserved DLco at the follow up visit, patients with DLco < 80% predicted had higher D-dimer levels at admission (*p* = 0.008), and higher TSS (*p* = 0.044) (Table 3 and Table 4). D-dimer was significantly correlated with DLco and VA both during hospitalization and at follow up (Figure 6). A multiple regression analysis was performed to predict DLco at follow up from FEV_1_, VC, TSS during hospitalization and D-dimer value at admission. The only variable that independently predicted DLco was D-dimer (*p* = 0.001), both when D-dimer was expressed as a continuous (log_10_ D-dimer) or a dichotomous variable (D-dimer > 1000 mg/L FEU) (Table 5). This result did not change when hospital or home treatment with LMWH was added to the model (Supplementary Materials, Table S3), or the highest D-dimer value reached during hospitalization was considered. Figure 7 and Figure 8 show an example of radiological and lung function evolution of two patients with impaired and normal DLco, respectively, during follow up.

## 4. Discussion

The main results of the present study can be summarized as follows: (1) during the acute phase patients with COVID-19 pneumonia showed a prevalent restrictive functional pattern, characterized by a concomitant reduction in VC and FEV_1_ and paralleled by an impairment in DLco; (2) the reduction in dynamic volumes and DLco was correlated with disease severity and was associated with the presence of lung consolidations both during the acute phase and after 6 weeks of follow up; (3) at 6 weeks post-discharge, DLco was at least mildly reduced in 65% of cases, while respiratory failure was completely resolved in all patients; and (4) D-dimer at admission was the only parameter that predicted the DLco value during the recovery phase.

Pulmonary sequelae in survivors from COVID-19 pneumonia have been a matter of intense discussion [16,34]. In fact, all studies, but one [12], performed a single lung functional assessment [9,10,11,13,14,15,16], limiting any hypothesis on the potential evolution and determinants of lung function impairment in these patients.

To our knowledge, this is the first study to show that, during the acute phase (about 9 days after hospital admission), about 50% of patients with COVID-19 pneumonia experience a restrictive respiratory pattern, with an equal and parallel reduction of FEV_1_, VC, and FVC, while only 10% of patients had a preserved DLco. The latter finding was sustained by a positive correlation with parenchymal consolidations at the chest CT and, in line with previous reports [12,16], it was directly associated with the fall in PaO_2_/FiO_2_ ratio observed in many patients. Indeed, testing patients just before hospital discharge, Fumagalli and colleagues [12] found a frequent reduction in FVC and FEV_1_, while Mo and coworkers [16] observed a restrictive disease in 25% and a reduced DLco in 47.2% of patients, a proportion that was doubled in case of severe disease.

After 6 weeks from hospital discharge, we observed a reduced DLco in 65% of patients, in line with the majority of available reports [10,13]. Contrary to previous studies [14], we did not find signs of airflow obstruction or small airways involvement in our cohort, probably due to the low prevalence of patients affected by chronic obstructive diseases and smoke exposure.

During follow up, ground glass opacities and septal fibrosis characterized >70% of patients, while only three subjects had a chest CT without any abnormality at the follow up visit. These data are in partial agreement with available reports. In fact, Rogliani and coworkers found almost no radiological abnormalities after 4 weeks of follow up in patients with mild to moderate COVID-19 pneumonia [10]. On the other hand, Shah and colleagues detected residual GGO in 83% of patients with moderate to critical disease [14]. Indeed, in contrast with our cohort, only 11% of the patients enrolled by Rogliani et al. had a severe respiratory failure, thus expecting the possibility of milder lung involvement and faster radiological restitutio ad integrum [10].

Some authors speculated that, in patients recovering from COVID-19 pneumonia, the observed reduction in DLco could be secondary to the development of fibrosis [11]. Unexpectedly, we did not find an association between DL_CO_ and lung fibrosis. Nevertheless, patients with a lower DLco at follow up had lower dynamic volumes, a higher prevalence of lung restriction (54% vs. 14%), and a worse TSS and higher D-dimer values during hospitalization compared with patients with preserved DLco.

We found that D-dimer at admission had a strong association with DLco and VA, and independently predicted DLco values during follow up. It is well recognized that patients with COVID-19 pneumonia are at high risk of micro and macro thrombotic events [3,35,36]. In the latter case, an increase in D-dimer would be expected to be related with KCO, an index of alveolar-capillary membrane dysfunction [37]. However, D-dimer showed only a weak correlation with KCO, and no association was found with in-hospital or home treatment with LMWH. We, therefore, hypothesize that the dynamics of DLco reduction and its relationship with D-dimer could be one or a combination of the following. First, a KCO overestimation due to the assessment of DLco in patients with a functional restrictive pattern [38] may have masked the possible persistence of a damage to the vascular component. Second, the reduction of static volumes, and hence the persistence of VA impairment during the recovery phase, excluding respiratory muscles contractile abnormalities, could be secondary to the lung anatomical derangement, which has been shown to persist up to 4–5 months after hospital discharge in patients with COVID-19 pneumonia [11]. Third, considering the low prevalence of patients with history of chronic lung diseases and obesity in our cohort, a re-distribution of the regional ventilation/perfusion ratio might have partially compensated the gas transfer efficiency, maintaining satisfactory KCO levels in some patients.

Despite the physiological and pivotal design of our study, we believe that these observations can provide useful hints from the clinical point of view. In fact, we suggest that during follow up, patients that survived a COVID-19 pneumonia should be monitored with lung function tests, particularly with DLco in case of higher D-dimer levels during hospitalization.

The present study has limitations. First, the small sample size, limited by the reduction of COVID-19 cases during May 2020 in our region. Second, the study enrolled patients with moderate-to-severe disease, limiting the generalizability of the study findings to milder forms of SARS_CoV-2 infection. Third, the lack of assessment of static volumes during the first evaluation might have caused an underestimation of the real number of restricted patients during the acute phase. Finally, the lack of lung function data in the period preceding the SARS-CoV-2 pandemic may have limited the estimation of the actual pathophysiological effects of COVID-19 pneumonia; indeed, the number of patients with such measurements would be very low, considered the very low prevalence of patients with chronic respiratory diseases in our sample.

## 5. Conclusions

In conclusion, for the first time, lung volumes and DLco in patients hospitalized with moderate to severe COVID-19 pneumonia have been assessed both during hospitalization and follow up. Patients acutely experience a mild to severe reduction in DLco that can persist up to 6 weeks post-discharge. Moreover, DLco impairment during follow up is predicted by D-dimer values at hospital admission, confirming the role of D-dimer as a marker of severity of lung involvement. In COVID-19 patients, radiological and functional parameters appear to follow different dynamics, and, currently, the proportion of patients that would suffer for permanent functional abnormalities (e.g., fibrosis) and which could be the effect of reduced DLco on exercise capacity or daily life activities remains largely unknown. Long-term follow up studies will be required to address this question.

## Figures and Tables

**Table 1:** Anthropometric, clinical characteristics, in-hospital treatments, and outcomes of patients included in the final analysis.

		Covid-19 Patients (*n*= 20)
Age at admission, years		58.2 (15.5)
Males, *n* (%)		14 (70)
BMI, kg/m^2^		27.6 (5.7)
Ex and active smokers, *n* (%)		4 (20%)
Arterial hypertension, *n* (%)		11 (55)
Diabetes mellitus, *n* (%)		2 (10)
Ischaemic heart disease, *n* (%)		2 (10)
Chronic treatments		
Angiotensin receptor blockers, *n* (%)		5 (20)
ACE inhibitors, *n* (%)		2 (10)
Blood count & Biochemistry at admission		
Lymphocytes < 1500 × 10^9^/µL, *n* (%)		14 (70)
D-dimer, mg/L FEU		768 (374–3966)
D-dimer ≥ 1000 mg/L FEU, *n* (%)		9 (45)
Gas exchange at admission		
pH		7.45 (0.03)
PaCO_2_, mmHg		42 (35–49)
PaO_2_/FiO_2_, mmHg		264 (128)
PaO_2_/FiO_2_	<100 mmHg, *n* (%)	2 (10)
	100–200 mmHg, *n* (%)	6 (30)
	201–300 mmHg, *n* (%)	4 (20)
	>300 mmHg, *n* (%)	8 (40)
A-a O_2_ gradient, mmHg		144 (17–253)
A-a O_2_ gradient >200 mmHg, *n* (%)		8 (40)
In-hospital treatments		
Systemic methylprednisolone, *n* (%)		15 (75)
LMWH, prophylaxis, *n* (%)		11 (55)
LMWH, anticoagulant, *n* (%)		9 (45)
CPAP, n (%)		13 (65)
CPAP duration, days		10.9 (3.5)
Outcomes		
From symptoms onset to 1st PFT, days		28.2 (13.5)
From admission to 1st PFT, days		9.1 (6.3)
From discharge to 2nd PFT, days		43.8 (5.7)
From 1st to 2nd PFT, days		52.4 (6.4)
Hospital stay duration, days		18.2 (11.7)
Treatments at discharge		
O_2_ therapy, *n* (%)		4 (20)
LMWH, *n* (%)		12 (60)

Data are reported as means (standard deviation, SD), medians (inter-quartile range, IQR) and frequencies. BMI = body mass index; LMWH = low molecular weight heparin; PFT = pulmonary function test; CPAP = continuous positive airway pressure; PEEP = positive end expiratory pressure; A-a O_2_ gradient = alveolar arterial oxygen gradient; PaO_2_/FiO_2_ = arterial partial oxygen pressure to fraction of inspired oxygen ratio; PaCO_2_ = arterial partial carbon dioxide pressure; INR = international normalized ratio; FEU = fibrinogen equivalent unit.

**Table 2:** Gas exchange and lung function during hospitalization and at the follow up visit.

	First PFT	Follow up PFT	Mean Difference (95% CI)	*p*-Value
Gas exchange				
FiO_2_, %	21 (21–32)	21 (21–21)	-	0.008
pH	7.45 (0.02)	7.44 (0.03)	−0.01 (−0.02–0.01)	0.436
PaCO_2_, mmHg	40 (32–43)	38 (34–42)	-	0.130
PaO_2_, mmHg	78 (71–88)	90 (87–100)	-	0.002
HCO3-, mEq/L	27.9 (3.1)	26.5 (2.7)	−1.4 (−3.0–0.2)	0.084
PaO_2_/FiO_2_, mmHg	316 (87)	443 (69)	126 (86–166)	<0.001
A-a O_2_ gradient, mmHg	34 (19–101)	9 (4–19)	-	<0.001
Spirometry				
FVC, liters	2.61 (1.01)	3.22 (1.07)	0.61 (0.34–0.88)	<0.001
FVC, % predicted	69.6 (15.9)	87.4 (16.0)	17.8 (10.0–25.6)	<0.001
VC, liters	2.73 (1.09)	3.35 (1.10)	0.62 (0.39–0.86)	<0.001
VC, % predicted	71.7 (16.9)	87.4 (16.2)	15.7 (9.9–21.5)	<0.001
FEV_1_, liters	2.22 (0.91)	2.69 (0.87)	0.46 (0.28–0.65)	<0.001
FEV_1_, % predicted	75.1 (18.9)	91.3 (15.2)	16.3 (8.9–23.6)	<0.001
FEV_1_/VC, % predicted	104.5 (98.2–114.0)	104.5 (98.2–111.0)	-	0.751
FEV_1_/VC < LLN, *n* (%)	3 (15)	0 (0)	-	0.250
Body-plethysmography*				
RV, liters	-	1.74 (0.50)	-	-
RV, % predicted	-	82.5 (28.8)	-	-
IC, liters	-	2.32 (0.71)	-	-
IC, % predicted	-	85.0 (23.5)	-	-
TLC, liters	-	5.19 (1.27)	-	-
TLC, % predicted	-	84.6 (15.6)	-	-
TLC < 80%predicted, *n* (%)	-	7 (35)	-	-
sRaw_tot_, kPa·s	-	0.83 (0.32)	-	-
sRaw_tot_, % predicted	-	75.0 (28.0)	-	-
Lung diffusion capacity				
DLco, mmol·min^−1^·kPa^−1^	4.98 (2.05)	5.95 (2.15)	0.96 (0.58–1.35)	<0.001
DLco, % predicted	56.0 (16.3)	67.2 (18.0)	11.2 (6.9–15.4)	<0.001
VA, liters	3.88 (1.34)	4.54 (1.38)	0.66 (0.39–0.92)	<0.001
VA, % predicted	64.8 (14.0)	75.3 (16.1)	10.5 (6.1–14.9)	<0.001
VC/VA	0.70 (0.12)	0.74 (0.07)	−0.04 (−0.1–0.03)	0.234
KCO, mmol·min^−1^·kPa^−1^·l^−1^	1.32 (0.36)	1.32 (0.28)	−0.00 (−0.09–0.92)	0.981
KCO, % predicted	89.1 (19.2)	91.7 (14.8)	2.65 (−3.2–8.5)	0.358

Data are reported as means (standard deviation, SD), medians (inter-quartile range, IQR) and frequencies. When appropriate, the 95% confidence interval (CI) is provided for the difference between follow up and hospitalization measurement. FEV_1_ = forced expiratory volume in one second; FVC = forced vital capacity; VC = vital capacity; LLN = lower limits of normal; RV = residual volume; IC = inspiratory capacity; TLC = total lung capacity; sRAWtot = specific total airway resistances; DLco = lung diffusion capacity for carbon monoxide; VA = alveolar volume; KCO = transfer factor for carbon monoxide. *Plethysmographic parameters were measured only at the follow up visit. Two patients were unable to perform the test (one patient had hearing loss, one patient was claustrophobic). The data reported for plethysmography are referred to 18 patients.

**Table 3:** Clinical characteristics during hospitalization in patients with DLco ≥80% predicted and DLco < 80% predicted.

		DLco < 80% Predicted (*n* = 13)	DLco ≥ 80% Predicted (*n* = 7)	*p*-Value
Age, years		58.5 (14.6)	57.7 (18.3)	0.921
BMI, Kg/m^2^		24.9 (23.3–27.2)	31.1 (22.7–33.0)	0.311
Males, *n* (%)		9 (69)	5 (71)	0.664
Arterial hypertension, *n* (%)		7 (54)	4 (57)	0.630
Diabetes mellitus, *n* (%)		2 (15)	0 (0)	0.411
Ischemic heart disease, *n* (%)		1 (8)	1 (14)	0.589
Biochemistry at admission				
White blood cells × 10^9^/µL		9420 (5010–14,515)	5890 (4560–7720)	0.347
D-dimer, mg/L FEU		3375 (607–5699)	394 (200–733)	**0.008**
D-dimer > 1000 mg/L FEU, *n* (%)		9 (69)	0 (0)	**0.004**
INR		1.25 (1.13–1.42)	1.17 (1.10–1.27)	0.516
Lymphocytes × 10^9^/µL		1194 (590)	1311 (726)	0.701
Lymphocytes, %		15.3 (9.9)	24.3 (15.3)	0.125
LDH, U/L		370 (151)	271 (127)	0.161
Interventions during hospitalization				
CPAP, *n* (%)		10 (77)	3 (43)	0.151
PEEP, mean (SD)		10.2 (1.5)	10.0 (2.5)	0.869
CPAP days, mean (SD)		11 (4)	11 (3)	0.957
LMWH, *n* (%)		13 (100)	6 (86)	0.350
LMWH, *n* (%)	Treatment	6 (46)	5 (71)	0.272
	Prophylaxis	7 (54)	1 (14)	0.106
Corticosteroids, *n* (%)		10 (77)	5 (71)	0.594

Data are expressed as means (SD), median (IQR) and frequencies, as appropriate. Significant differences are in bold. BMI = body mass index; LMWH = low molecular weight heparin; CPAP = continuous positive airway pressure; PEEP = positive end expiratory pressure; INR = international normalized ratio; FEU = fibrinogen equivalent unit.

**Table 4:** Clinical and functional characteristics during hospitalization and at follow up in patients with DLCO ≥ 80% predicted and DLCO < 80% predicted.

	DL_CO_ ≥ 80% Predicted (*n* = 13)		DL_CO_ < 80% Predicted (*n* = 7)			
	During Hospitalisation	Follow up *	During Hospitalisation	Follow up *	*p*-Value	*p*-Value *
Chest CT patterns						
TSS, points	10.2 (6.0)	6.4 (4.0)	7.4 (3.5)	6.0 (3.3)	**0.044**	0.797
Peripheral GGO, n (%)	9 (69)	5 (38)	5 (71)	4 (57)	0.664	0.370
Multifocal GGO, n (%)	5 (38)	10 (77)	1 (14)	6 (86)	0.277	0.561
Crazy paving, n (%)	4 (31)	1 (8)	2 (29)	0 (0)	0.664	0.650
Consolidation, n (%)	9 (69)	2 (15)	4 (57)	0 (0)	0.474	0.411
Fibrosis, n (%)	8 (61)	10 (77)	4 (57)	4 (57)	0.608	0.336
Post vs. ant. Prev., n (%)	10 (77)	10 (77)	6 (86)	6 (86)	0.561	0.561
Gas exchange parameters						
FiO_2_, %	24 (21–32)	21 (0)	21 (21–35)	21 (0)	0.316	*n*/a
pH	7.47 (7.44–7.48)	7.45 (7.42–7.47)	7.43 (7.42–7.44)	7.44 (7.41–7.45)	0.853	0.211
PaCO_2_, mmHg	39.8 (4.6)	37.8 (4.1)	42.1 (8.0)	39.4 (5.5)	0.404	0.457
PaO_2_, mmHg	76.5 (8.3)	89.8 (13.1)	83.1 (12.4)	99.1 (15.9)	0.169	0.173
HCO3^-^, mmol/L	28.3 (2.6)	26.5 (2.2)	27.2 (4.0)	26.4 (3.6)	0.452	0.933
PaO_2_/FiO_2_, mmHg	300 (74)	427 (62)	343 (109)	472 (76)	0.325	0.173
A-a O_2_ gradient, mmHg	38 (25–108)	12 (6–20)	18 (16–99)	0.0 (0–0)	0.126	0.056
Lung function						
FVC, %predicted	63.1 (12.1)	82.8 (15.7)	81.6 (15.8)	95.5 (13.8)	**0.023**	0.082
FEV_1_, %predicted	67.6 (15.1)	86.5 (15.8)	88.6 (18.1)	100.4 (9.2)	**0.012**	**0.047**
VC, %predicted	62.6 (11.8)	82.8 (17.3)	88.6 (10.6)	96.1 (9.8)	**<0.001**	0.077
FEV_1_/VC, %predicted	105.8 (18.5)	105.1 (10.1)	106.4 (13.6)	104.7 (9.1)	0.935	0.935
TLC, %predicted	-	80.0 (15.5)	-	91.9 (13.7)	-	0.119
TLC >80%predicted, n (%)		6 (54.5)		1 (14.3)		0.112
IC, %predicted	-	78.0 (23.1)	-	96.0 (21.2)	-	0.116
RV, %predicted	-	74.8 (24.5)	-	94.6 (32.6)	-	0.162
DLco, %predicted	48.3 (14.2)	57.5 (14.1)	70.2 (8.4)	85.1 (6.6)	**0.002**	**<0.001**
KCO, %predicted	83.5 (20.5)	86.4 (14.7)	98.6 (12.5)	101.8 (9.1)	0.096	**0.022**
VA, %predicted	60.6 (13.4)	69.4 (16.1)	72.7 (12.3)	86.4 (8.9)	0.064	**0.020**
VA/TLC		0.87 (0.13)		0.92 (0.1)		0.394
VA/VC	0.67 (0.12)	0.75 (0.06)	0.75 (0.09)	0.70 (0.08)	0.159	0.102

Data are expressed as means (SD), median (IQR) and frequencies, as appropriate. Significant differences are in bold. TSS = total severity score; GGO = ground glass; A-a O_2_ gradient = alveolar arterial oxygen gradient; PaO_2_/FiO_2_ = arterial partial oxygen pressure to fraction of inspired oxygen ratio; PaCO_2_ = arterial partial carbon dioxide pressure; FEV_1_ = forced expiratory volume in one second; FVC = forced vital capacity; VC = vital capacity; LLN = lower limits of normal; RV = residual volume; IC = inspiratory capacity; TLC = total lung capacity; sRAWtot = specific total airway resistances; DLco = lung diffusion capacity for carbon monoxide; VA = alveolar volume; KCO = transfer factor for carbon monoxide.*comparison between the follow up observations.

**Table 5:** Predicting factors for DLco in the post-discharge period.

	Unstandardized Coefficient	Standardized Coefficient	95% CI	*p*-Value
Model 1 (adj R^2^: 0.786)				
FEV_1_ %pred	−0.043	−0.050	−0.442–0.355	0.820
VC %pred	0.227	0.235	−0.294–0.748	0.368
TSS, points	−0.623	−0.155	−1.877–0.632	0.307
Lg_10_ D-dimer	−18.675	−0.655	−28.373– −9.076	0.001
Model 2 (adj R^2^: 0.786)				
FEV_1_ %pred	−0.026	−0.030	−0.426–0.373	0.868
VC %pred	0.161	0.167	−0.374–0.697	0.172
TSS, points	−0.426	−0.106	−1.710–0.859	0.209
D-dimer ≥ 1000 FEU	−22.849	−0.716	−34.594– −11.105	0.001

Multiple regression analysis models for predicting DLco % predicted at follow up. Having a D-dimer > 1000 at admission is associated with a DLco reduction of 22% at 6 weeks post-discharge. Variables significantly different at the univariate analysis (FEV_1_, VC, TSS, and D-dimer) were used for the linear regression analysis. FVC was excluded from the model because it is intrinsically highly correlated with VC (see legend of Figure 4 for details). CI = confidence interval; FEV_1_ = forced expiratory volume in one second; VC = vital capacity; TSS = total severity score

## Data Availability

The anonymized datasets used and analyzed during the current study are available from the corresponding Author on reasonable request.

## Supplementary Materials

The following are available online at https://www.mdpi.com/2077-0383/10/5/1021/s1, Table S1. Radiological patterns during the acute phase and after 6 weeks post-discharge, Table S2. Relationship between lung function parameters and chest CT patterns during the acute phase and at 6 weeks post-discharge, Table S3. Multiple regression analysis for predicting DLCO %predicted at follow up, Table S4. STROBE 2007 (v4) Statement—Checklist of items that should be included in reports of cohort studies, Supplementary Materials and Methods.
