# Supplementary material for: Trends over Time of Lung Function and Radiological Abnormalities in COVID-19 Pneumonia: A Prospective, Observational, Cohort Study
Source: J Clin Med. 2021 Mar 2;10(5):1021. doi: 10.3390/jcm10051021 (PMC7958849; doi:10.3390/jcm10051021)
Supplement: Supplementary file 1 [file jcm-10-01021-s001.pdf]

Supplementary materials

Table S1. Radiological patterns during the acute phase and after 6 weeks post-discharge.

Chest CT Scores	First CT	Follow up CT	Mean Diff. (95% CI)	*p*-Value
pGGO, *n* (%)	14 (70)	9 (45)	-	0.180
mGGO, *n* (%)	6 (30)	16 (80)	-	0.002
Crazy paving, *n* (%)	6 (30)	1 (5)	-	0.125
Consolidation, *n* (%)	13 (65)	2 (10)	-	0.001
Fibrosis, *n* (%)	12 (60)	14 (70)	-	0.754
Posterior vs anterior prevalence, *n* (%)	16 (80)	13 (65)	-	0.375
TTS	7.9 (4.0)	6.3 (3.7)	1.6 (-3.7–0.46)	0.118
TSS improvement ≥ 2 points	-	8 (40)		n/a

pGGO = peripheral ground glass opacities; mGGO = multifocal ground glass opacities; TTS = total severity score.

Table S2. Relationship between lung function parameters and chest CT patterns during the acute phase and at 6 weeks post-discharge.

	During Hospitalisation									
	**pGGO**	***p*-Value**	**mGGO**	***p*-Value**	**Crazy Paving**	***p*-Value**	**Consolidation**	***p*-Value**	**Fibrosis**	***p*-Value**
FEV1 %pred	0.186	0.216	0.049	0.418	0.055	0.409	-0.323	0.082	-0.048	0.421
VC %pred	0.154	0.259	-0.306	0.095	-0.247	0.147	-0.345	0.068	-0.027	0.455
DLCo %pred	0.220	0.176	-0.179	0.226	-0.014	0.477	**-0.423**	**0.032**	-0.199	0.200
Kco %pred	-0.002	0.496	0.044	0.427	0.072	0.381	-0.114	0.317	0.037	0.438
VA %pred	0.312	0.090	-0.240	0.154	-0.112	0.319	**-0.506**	**0.011**	-0.307	0.094
VA/VC	-0.168	0.239	**-0.370**	**0.054**	**-0.459**	**0.021**	-0.153	0.260	0.126	0.298
	**At Follow up**									
	**pGGO**	***p*-Value**	**mGGO**	***p*-Value**	**Crazy Paving**	***p*-Value**	**Consolidation**	***p*-Value**	**Fibrosis**	***p*-Value**
FEV1 %pred	-0.245	0.299	-0.064	0.789	-0.160	0.501	**-0.580**	**0.007**	-0.366	0.112
VC %pred	-0.242	0.303	0.125	0.599	-0.166	0.483	**-0.664**	**0.001**	-0.285	0.222
DLCo %pred	0.051	0.832	0.398	0.082	0.024	0.921	**-0.489**	**0.029**	-0.273	0.244
Kco %pred	0.358	0.121	0.391	0.089	0.116	0.628	0.284	0.226	-0.057	0.812
VA %pred	-0.308	0.187	0.202	0.393	-0.063	0.790	**-0.728**	**<0.001**	-0.284	0.225
VA/VC	-0.193	0.415	-0.231	0.328	0.196	0.408	0.015	0.949	-0.249	0.291

Pearson's correlation coefficients are shown. Significant correlations are in bold. FEV1 = forced expiratory volume in one second; VC = vital capacity; DLCO = lung diffusion capacity for carbon monoxide; VA = alveolar volume; KCo = transfer factor for carbon monoxide; pGGO = peripheral ground glass opacities; mGGO = multifocal ground glass opacities; %pred = % predicted value.

Table S3. Multiple regression analysis for predicting DLCO %predicted at follow up.

	Unstandardized coefficient	Standardized coefficient	95% CI	*p*-Value
Model 3<br>(adj R^2: 0.735)				
FEV1 %pred	-0.037	-0.043	-0.436 – 0.361	0.843
VC %pred	0.101	0.105	-0.444 – 0.646	0.696
TTS, points	-0.444	-0.111	-1.724 – 0.835	0.469
D-dimer ≥ 1000 FEU	-23.297	-0.730	-36.020 – -11.573	0.001
Any LMWH	-10.904	-0.150	-31.251 – -11.573	0.270
Model 4<br>(adj R^2: 0.717)				

FEV1 %pred	-0.027	-0.032	-0.438 – 0.384	0.889
VC %pred	0.149	0.258	-0.404 – 0.702	0.572
TTS, points	-0.497	-0.124	-1.844 – 0.849	0.441
D-dimer≥1000 FEU	-24.156	-0.757	-37.123 – -11.189	0.001
Therapeutic LMWH	2.765	0.085	-7.206 – 12.736	0.561
Model 5<br>(adj R^2: 0.726)				
FEV1 %pred	-0.076	0.088	-0.498 – 0.346	0.705
VC %pred	0.177	0.183	-0.367 – 0.720	0.497
TTS, points	-0.426	0.603	-1.644 – 1.009	0.616
D-dimer ≥ 1000 FEU	-24.194	-0.758	-36.513 – -11.875	0.001
Home LMWH	-3.872	-0.119	-13.106 – -5.362	0.384

The models reported include LMWH (any LMWH during hospitalisation, therapeutic LMWH regimen during hospitalization and home treatment with LMWH) in addition to FEV1, VC, TTS and having a D-dimer >1000 mg/L FEU at admission. FVC was excluded from the model because it was pathophysiologically highly correlated with VC (see legend of Figure 4 in the main text for details). CI = confidence interval; FEV1 = forced expiratory volume in one second; FVC = forced vital capacity; LMWH = low molecular weight heparin; VC = vital capacity; TTS = total severity score.

Table S4. STROBE 2007 (v4) Statement—Checklist of items that should be included in reports of cohort studies

**Section/Topic**	**Item** #	**Recommendation**	**Reported on page** #
**Title and abstract**	1	(*a*) Indicate the study's design with a commonly used term in the title or the abstract	1
		(*b*) Provide in the abstract an informative and balanced summary of what was done and what was found	2
**Introduction**			
Background/rationale	2	Explain the scientific background and rationale for the investigation being reported	4
Objectives	3	State specific objectives, including any prespecified hypotheses	4
**Methods**			
Study design	4	Present key elements of study design early in the paper	5
Setting	5	Describe the setting, locations, and relevant dates, including periods of recruitment, exposure, follow-up, and data collection	5
Participants	6	(*a*) Give the eligibility criteria, and the sources and methods of selection of participants. Describe methods of follow-up	5
		(*b*) For matched studies, give matching criteria and number of exposed and unexposed	n/a
Variables	7	Clearly define all outcomes, exposures, predictors, potential confounders, and effect modifiers. Give diagnostic criteria, if applicable	5,6
Data sources/ measurement	8*	For each variable of interest, give sources of data and details of methods of assessment (measurement). Describe comparability of assessment methods if there is more than one group	6,7
Bias	9	Describe any efforts to address potential sources of bias	8
Study size	10	Explain how the study size was arrived at	8,9

Quantitative variables	11	Explain how quantitative variables were handled in the analyses. If applicable, describe which groupings were chosen and why	8,9
Statistical methods	12	(*a*) Describe all statistical methods, including those used to control for confounding	8,9
		(*b*) Describe any methods used to examine subgroups and interactions	8,9
		(*c*) Explain how missing data were addressed	8,9
		(*d*) If applicable, explain how loss to follow-up was addressed	n/a
		(*e*) Describe any sensitivity analyses	n/a
**Results**			
Participants	13*	(a) Report numbers of individuals at each stage of study—eg numbers potentially eligible, examined for eligibility, confirmed eligible, included in the study, completing follow-up, and analysed	9
		(b) Give reasons for non-participation at each stage	9
		(c) Consider use of a flow diagram	9 (Figure 1)
Descriptive data	14*	(a) Give characteristics of study participants (eg demographic, clinical, social) and information on exposures and potential confounders	Table 1
		(b) Indicate number of participants with missing data for each variable of interest	n/a
		(c) Summarise follow-up time (eg, average and total amount)	9
Outcome data	15*	Report numbers of outcome events or summary measures over time	12-17
Main results	16	(*a*) Give unadjusted estimates and, if applicable, confounder-adjusted estimates and their precision (eg, 95% confidence interval). Make clear which confounders were adjusted for and why they were included	12-17
		(*b*) Report category boundaries when continuous variables were categorized	n/a
		(*c*) If relevant, consider translating estimates of relative risk into absolute risk for a meaningful time period	n/a

Other analyses	17	Report other analyses done—eg analyses of subgroups and interactions, and sensitivity analyses	12-17
**Discussion**			
Key results	18	Summarise key results with reference to study objectives	18
**Limitations**			20
Interpretation	20	Give a cautious overall interpretation of results considering objectives, limitations, multiplicity of analyses, results from similar studies, and other relevant evidence	18,19
Generalisability	21	Discuss the generalisability (external validity) of the study results	19
**Other information**			
Funding	22	Give the source of funding and the role of the funders for the present study and, if applicable, for the original study on which the present article is based	n/a

*Give information separately for cases and controls in case-control studies and, if applicable, for exposed and unexposed groups in cohort and cross-sectional studies.

Note: An Explanation and Elaboration article discusses each checklist item and gives methodological background and published examples of transparent reporting. The STROBE checklist is best used in conjunction with this article (freely available on the Web sites of PLoS Medicine at http://www.plosmedicine.org/, Annals of Internal Medicine at http://www.annals.org/, and Epidemiology at http://www.epidem.com/). Information on the STROBE Initiative is available at www.strobe-statement.org.

Supplementary Materials and Methods

COVID-19 diagnosis

The diagnosis of COVID-19 pneumonia was based on a positive nasopharyngeal swab for SARS-CoV-2 collected in the emergency department and on the presence of typical pulmonary infiltrates at the chest X-ray or CT scan [1,2]. The SARS-CoV-2 infection was confirmed by means of reverse transcriptase PCR (RT-PCR). The presence of viral, bacterial, or fungal co-infections and alternative diagnoses were also excluded, as previously reported [1].

Gas exchange parameters

Following ten minutes of rest, while in seated position, an arterial blood sample was obtained from each patient and processed with a GEM Premier 5000 gas analyzer (Instrumentation Laboratory, Lexington, MA, USA). Patients, at the time of the test, could be on oxygen therapy. The following gas exchange parameters were obtained: pH, arterial partial pressure of oxygen (PaO2), arterial partial pressure of carbon dioxide (PaCO2), oxygen saturation (SaO2), and the PaO2 to fraction of inspired oxygen (PaO2/FiO2) ratio. The presence of respiratory failure was defined as a PaO2/FiO2 < 300 mmHg, and was graded as follows: mild (PaO2/FiO2 201–300 mmHg), moderate (PaO2/FiO2 101–200 mmHg), and severe (PaO2/FiO2 ≤ 100 mmHg) [1].

Lung function testing

A moving cart equipped with a spirometer and a lung diffusion analyzer (Quark PFT, Cosmed, Roma, Italy) was moved into the HDRU between April and May 2020. While seated in a wheelchair, patients underwent the measurement of slow (VC) and forced (FVC) vital capacity, forced expiratory volume in one second (FEV1), FEV1/VC ratio, lung diffusion capacity for carbon monoxide (DLco), alveolar volume (VA), and transfer factor (KCO). At the follow up visit, static volumes (residual volume—RV; intra-thoracic gas volume—ITGV; total lung capacity—TLC) and specific total airway resistances (sRAWtot) were assessed by means of a constant-volume body plethysmograph (MasterScreen Body; Erich Jaeger GmbH, Würzburg, Germany). ITGV was obtained at functional residual capacity and subtracted from TLC to calculate RV, while sRAWtot were measured during tidal breathing.

Management of Respiratory Failure

Helmet continuous positive airway pressure (CPAP) was initiated when patients showed peripheral oxygen saturation (SpO2) values $< 94\%$ with a Reservoir mask at 90–100% FiO2 or showed sign of respiratory distress [2,3,4]. Positive end expiratory pressure (PEEP) was titrated based on recruitment, hemodynamic stability, comorbidities, and respiratory distress, and set to a maximum of 10 cmH2O, according to local standard operating procedures and national and international consensus statements [3,5,6]. Patients that failed a CPAP trial were evaluated by the Intensive Care Unit (ICU) staff and by the treating attending physician for potential intubation or to establish a do not intubate order, considering patients' probability of hospital and ICU survival, comorbidities, and fragility score, as previously reported [2,3].

Pharmacological therapy

According to local standard operating procedures and available recommendations [7], unless contraindicated, patients were administered hydroxychloroquine, lopinavir/ritonavir, and off-label immunomodulation with tocilizumab. Prophylactic low molecular weight heparin (LMWH) was administered to all patients at risk of deep vein thrombosis (DVT), while therapeutic dosages were given in case of confirmed DVT or pulmonary embolism, critically ill patients, or when the D-dimer value was > 3000 FEU. Systemic methylprednisolone was administered in patients with severe pneumonia as recommended by ATS guidelines on community acquired pneumonia [8]. When indicated, patients continued LMWH after hospital discharge for at least 15 days. Clinically stable patients with persistent respiratory failure (PaO2 < 60 mmHg) in room air were discharged home with long-term oxygen therapy.

Chest CT methodology and interpretation

The parameters used for the scanning protocols were as follows: patients in supine position; endinspiratory acquisition; tube voltage: 120–140 kVp; automatic tube current modulation: 100–300 mAs; pitch: 0.5; section thickness after reconstruction: 1.25 mm. Unenhanced CT scans were obtained for all patients.

Two experienced radiologists (N.F. and S. I.) with 20 and 15 years of experience in thoracic radiology and with a broad expertise in the identification of COVID-19 radiological patterns retrospectively and independently reviewed the images on a PACS work-station (IMPAX, Agfa Healthcare) with multiplanar reconstructions tools and reached a shared decision by consensus. Chest CT images were assessed for the presence of peripheral and multifocal ground glass opacities, consolidations, fibrosis, and crazy-paving pat-terns. The severity of disease was evaluated using the Total Severity Score

(TSS), a quantitative CT scoring system with good intraclass correlation and inter-observer reproducibility [9], developed by Kunwei and colleagues in March 2020 [9,10]. To assess the TSS, each of the five pulmonary lobes was assessed for the degree of involvement and classified from 0 to 4 depending on the extension of the lobe involvement: 0 (0%), 1 (1–25%), 2 (26–50%), 3 (51–75%), or 4 (76–100%). An overall lung total severity score was reached by summing the five lobe scores (range of possible scores, 0–20).

REASONS FOR DROP-OUT

Twenty-seven patients were enrolled in the study, and 7 were lost at the follow up visit. Two patients were still hospitalized in rehabilitation units, 2 did not answer the phone or were untraceable, 2 were too far from the hospital to come to the follow up visit, and 1 patient continued the follow up in another center.

REFERENCES

1. Santus, P.; Radovanovic, D.; Saderi, L.; Marino, P.; Cogliati, C.; De Filippis, G.; Rizzi, M.; Franceschi, E.; Pini, S.; Giuliani, F.; Del Medico, M.; Nucera, G.; Valenti, V.; Tursi, F.; Sotgiu, G. Severity of respiratory failure at admission and in-hospital mortality in patients with COVID-19: a prospective observational multicentre study. BMJ Open 2020, 10, e043651.
2. Aliberti, S.; Radovanovic, D.; Billi, F.; Sotgiu, G.; Costanzo, M.; Pilocane, T.; Saderi, L.; Gramegna, A.; Rovellini, A.; Perotto, L.; Monzani, V.; Santus, P.; Blasi, F. Helmet CPAP treatment in patients with COVID-19 pneumonia: a multicentre cohort study. Eur Respir J 2020, 56, 2001935.
3. Santus, P.; Radovanovic, D.; Saderi, L.; Marino, P.; Cogliati, C.; De Filippis, G.; Rizzi, M.; Franceschi, E.; Pini, S.; Giuliani, F.; Del Medico, M.; Nucera, G.; Valenti, V.; Tursi, F.; Sotgiu, G. Severity of respiratory failure at admission and in-hospital mortality in patients with COVID-19: a prospective observational multicentre study. BMJ Open 2020, 10, e043651.
4. Radovanovic, D.; Rizzi, M.; Pini, S.; Saad, M.; Chiumello, D.A.; Santus, P. Helmet CPAP to treat acute hypoxemic respiratory failure in patients with COVID-19: a management strategy proposal. J Clin Med 2020, 9, 1191.
5. Vitacca, M.; Nava, S.; Santus, P.; Harari, S. Early consensus management for non-ICU acute respiratory failure SARS-CoV- 2 emergency in Italy: from ward to trenches. Eur Respir J 2020, 55, 2000632.
6. Bassetti, M.; Giacobbe, D.R.; Aliberti, S.; Barisione, E.; Centanni, S.; De Rosa, F.G.; Di Marco, F.; Gori, A.; Granata, G.; Mikulska, M.; Petrosillo, N.; Richeldi, L.; Santus, P.; Tascini, C.; Vena, A.; Viale, P.; Blasi, F.; Italian Society of Anti-infective Therapy (SITA) and the Italian Society of Pulmonology (SIP). Balancing evidence and frontline experience in the early phases of the COVID-19 pandemic: current position of the Italian Society of anti-infective therapy (SITA) and the Italian Society of pulmonology (SIP). Clin Microbiol Infect 2020, 26, 880–94.
7. SIMIT (Società Italiana di Malattie Infettive e Tropicali). In: Sezione Regione Lombardia. Vademecum per la cura delle persone con malattia da COVID-19. 2.0 ed 2020. Available online: http://www.simit.org/medias/1569-covid19-vademecum-13-03-202.pdf (accessed on 18 November 2020).
8. Metlay, J.P.; Waterer, G.W.; Long, A.C.; Anzueto, A.; Brozek, J.; Crothers, K.; Cooley, L.A.; Dean, N.C.; Fine, M.J.; Flanders, S.A.; Griffin, M.R.; Metersky, M.L.; Musher, D.M.; Restrepo, M.I.; Whitney, C.G.. Diagnosis and treatment of adults with community-acquired pneumonia. An official clinical practice guideline of the American Thoracic Society and Infectious Diseases Society of America. Am J Respir Crit Care Med 2019, 200, e45–e67.
9. Wasilewski, P.G.; Mruk, B.; Mazur, S.; Półtorak-Szymczak, G.; Sklinda, K.; Walecki, J. COVID-19 severity scoring systems in radiological imaging - a review. *Pol J Radiol* **2020**, 85, e361-e368.
10. Li, K.; Fang, Y.; Li, W.; Pan, C.; Qin, P.; Zhong, Y.; Liu, X.; Liao, Y.; Li, S. CT image visual quantitative evaluation and clinical classification of coronavirus disease (COVID-19). *Eur Radiol* **2020**, 30, 4407-16.
